# Associations of in-hospital postpartum feeding experiences with exclusive breastfeeding practices among infants in rural Sichuan, China

**DOI:** 10.1186/s13006-023-00567-z

**Published:** 2023-07-13

**Authors:** Ann M. Weber, Yian Guo, Evelyn Zhang, Susan Gruber, Alexis Medina, Huan Zhou, Gary L. Darmstadt

**Affiliations:** 1 Department of Biostatistics, Epidemiology and Environmental Health, School of Public Health, University of Nevada, Reno, NV USA; 2 Rural Education Action Program (REAP), Freeman Spogli Institute for International Studies, Stanford University, Stanford, CA USA; 3 Putnam Data Sciences, LLC, Cambridge, MA USA; 4 Department of Health and Social Behavior Science, West China School of Public Health, Sichuan University, No.16, Section 3, South Renmin Road, Chengdu City, Sichuan Province 610041 People’s Republic of China; 5 Department Pediatrics, Stanford University School of Medicine, Stanford, CA USA

**Keywords:** Exclusive breastfeeding, Infant and young child feeding, Infant formula, Rural China, Baby-friendly hospitals, Public health policy

## Abstract

**Background:**

In rural China, exclusive breastfeeding (EBF) prevalence is low and hospitals often fail to attain baby-friendly feeding objectives, such as ≥ 75% of newborns exclusively breastfed from birth to discharge. Empirical evidence for the impact of increased hospital compliance with recommended feeding guidelines on continued EBF in rural China is lacking. We sought to measure and model the association of newborns’ in-hospital feeding experiences with EBF practice in infancy to inform policies for EBF promotion.

**Methods:**

Data were cross-sectional from 785 caregivers of infants < 6 months of age, collected from November to December 2019 in four underdeveloped counties/districts in Sichuan Province. In-hospital feeding practices were determined, and prevalence of current infant feeding practices was calculated from 24-h recall and categorized according to WHO/UNICEF Infant and Young Child Feeding categories as EBF, breastfed with non-milk liquids, mixed feeding, breastfed with solids, and not breastfed. Relative risk ratios were estimated using adjusted multinomial logistic regression to examine risk factors for non-EBF practices compared to EBF, including in-hospital feeding experiences. The regression model was used to investigate change in EBF prevalence under alternative in-hospital experiences.

**Results:**

Only 38.1% of under-six-month-old infants were being exclusively breastfed when data were collected; 61.8% and 77.6% had been fed water and infant formula, respectively, in the hospital. Infants who were fed water or formula before discharge were estimated as 2–3 times as likely to be non-EBF than EBF up to age six months. According to our model, EBF prevalence would have increased to 53.7% (95% confidence interval (CI) 46.1, 61.2) had ≥ 75% of infants been exclusively breastfed and water-based feeds eliminated in-hospital.

**Conclusions:**

Given the importance of infants’ first feeding experiences in the establishment and continuation of EBF, it is imperative that rural Chinese hospitals actively seek to limit infant formula feeds to medically indicated situations and eliminate water-based feeds.

**Supplementary Information:**

The online version contains supplementary material available at 10.1186/s13006-023-00567-z.

## Background

In a recent study in China, less than 30% of infants under six months of age were found to be exclusively breastfeeding [1]; EBF rates were < 10% in some rural areas [2, 3]. In contrast, the infant formula market is rapidly expanding in middle-income countries like China [4], with almost 45% of rural Chinese newborns receiving formula before leaving the hospital [2]. Although China has made increasing EBF prevalence a national priority [5], it is unclear which interventions should be prioritized, especially in rural contexts challenged by poverty, traditional practices, and lack of support for EBF.

Prior research has identified many barriers to EBF in LMICs. While maternal characteristics, including returning to paid employment and rural-to-urban migration [6], have been shown to influence breastfeeding decisions [7–9], in-hospital postpartum experiences are also predictive of later infant feeding practices. Infants who receive formula in the hospital are less likely to be exclusively breastfed for six months [7, 8, 10], whereas breastfeeding initiation within the first hour after birth [1, 7] and providing breastmilk at the first feeding [11] are positively associated with later EBF. In China, pre-lacteal feeds before breastfeeding initiation are a common, and possibly traditional [12] practice; 26–93% of infants are given something other than breastmilk as their first feed [13–16]. Importantly, 20–30% of Chinese newborns are first fed water [13, 15, 16], despite the lack of any medical indication for the practice. Data specifically from rural areas of China are limited, but indicate that only 41% of infants were first fed breastmilk [11] and as few as 9% of rural mothers initiated breastfeeding within one hour of childbirth [15].

To reinforce optimal in-hospital feeding practices globally, UNICEF and WHO launched the Baby-Friendly Hospital Initiative (BFHI) in 1991, recommending, for example, that ≥ 75% of newborns should be exclusively fed breastmilk from birth to discharge [17]. Programs that provide BFHI training to health workers and target immediate postpartum feeding experiences have been shown to increase EBF across diverse LMIC settings [18, 19], including urban China [20]. To our knowledge, no such studies have been performed in rural China despite low EBF prevalence. The aims of this study were to measure and model the associations of sub-optimal hospital feeding practices with continued EBF to six months in a rural Chinese province, and to estimate the potential effect of achieving the BFHI target of ≥ 75% EBF among newborns at discharge on EBF prevalence during the first six months.

## Methods

This study used cross-sectional data collected at baseline for a randomized controlled trial (RCT) of a home-visiting program planned for implementation in rural Sichuan Province, China. Nearly 40% of Sichuan’s population are rural residents, with an average annual per capita disposable income of RMB 13,331 ($1906), less than half of the national average of RMB 28,228 ($4033) [21]. Due to national shutdowns across China to control the spread of COVID-19 within weeks of baseline data collection, the RCT was not implemented.

### Sampling

Sampling of participants was multi-stage. In Stage 1, 80 townships were selected using stratified random sampling from four previously designated national “poverty counties” (*pinkun xian*) of Sichuan (20 townships per county). Non-rural townships were excluded from the sampling frame because urban-dwelling families have access to better schools, social welfare programs, and health care compared to those in rural areas. Moreover, formative field work indicated that families in rural areas are at higher risk for poor maternal and child health outcomes. Rural townships with fewer than 10,000 people were also excluded to ensure adequate statistical power for the RCT. In Stage 2, a list provided by the county-level Maternal and Child Hospital was used to randomly sample 25 households per town from households with either: (1) pregnant women in their second or third trimester or (2) families with infants under seven months of age. If the town did not have 25 eligible households, the search radius was expanded to include villages in the township up to 60 min away from the town. This strategy identified 357 households with pregnant women and 939 households with infants, for a total of 1,296 households. For this study, we included 830 households with an infant under age six months, excluding 109 households with children ages six months and above. We also excluded infants from the analysis with missing age (*n* = 2) or missing primary outcome information (*n* = 3), who were not breastfed due to medical reasons (*n* = 4), or whose primary caregiver was not the biological mother (*n* = 36), for a final analytic sample of 785. Consistent with the last two criteria, excluded caregivers were more likely to be older (e.g., a grandmother) and excluded children were more likely to have been born premature and received formula as their first feed (and no colostrum) (Table S4). Excluded children were also more likely to be older, female, and have wealthier families.

### Data collection

Data were collected in face-to-face interviews from November to December 2019. The primary outcome was infants’ current EBF status, based on a 24-h dietary recall [22] of infant consumption of breastmilk, breastmilk substitutes (e.g., infant formula or animal milk), water, non-milk liquids (e.g., juice or broth), and semi-solid or solid foods (e.g., porridge or rice), as described in Table S1 in the Supplemental Materials. A current feeding practice variable was generated from these data consisting of five mutually exclusive feeding groups based on the WHO/UNICEF Infant and Young Child Feeding categories [22]: 1) breastmilk only (EBF); 2) breastmilk and non-milk liquids; 3) mixed feeding of breastmilk and animal milk or formula; 4) breastmilk and solid or semi-solid foods; and 5) not breastfed during the previous day (Table S2). WHO/UNICEF categories for breastfed with water only and breastfed with non-milk liquids were combined because only four infants received any non-milk liquid besides water in these two groups.

Information that was collected on in-hospital newborn feeding practices which were expected to be associated with continued EBF to six months included: if the infant was put to the breast within one hour of birth (indicating early initiation) [22], the composition of the infant’s first feed (“What was the child fed first after birth?”), and whether the infant was fed colostrum, water, sugar water, or formula at any point in the hospital (“Was the baby fed [water/sugared water/formula] at any time in the hospital?”). Mothers were also asked if they received infant formula samples while in the hospital or health center (Table S3). Data on whether the infant was “ever breastfed” were collected but not included in analyses as nearly all mothers (97%) in our sample reported that they had breastfed their infant. Questions on types of feeds given in-hospital were based on study authors’ prior research.

Infant and birth-related information was collected on infant age, sex, childbirth method [cesarean section (C-section) or vaginal delivery], preterm birth, and low birth weight status. Data collected on family characteristics included maternal age and education (categorized as primary, secondary, or tertiary education and above), wealth quintiles based on household assets [23], whether the mother married from outside the village where she was residing, and whether she resided in a town or village. Mothers’ previous migration for work was our proxy for possible future migration that could influence her decision to breastfeed [6]. Information on the type of hospital used for childbirth (e.g., township health center or maternal child health hospital) was also collected, but hospital name and baby-friendly status were not.

### Statistical analysis

Descriptive statistics for hospital feeding experiences and infant and demographic characteristics were calculated overall, by current feeding group, and by type of birthing hospital for the analytic sample of 785 mother–child dyads. Next, adjusted multinomial logistic (mlogit) regression [24] was used to model the association of these same factors with current feeding group membership. An extension of logistic regression, mlogit is used when the dependent variable has more than two unordered categories. In our analysis, EBF was selected as the reference category for comparison with the other current feeding groups. The mlogit model estimated a different set of coefficients for each of the non-EBF groups relative to EBF, which, when exponentiated, have a natural interpretation as relative risk ratios (RRR) [24] (see additional information on mlogit in the Supplemental Materials). For the regressions, preterm birth and low birth weight were combined into a single indicator as these were highly correlated (*r* = 0.80). Hospital type was collapsed to three categories (township, county or city-level) for parsimony (model fit was not significantly improved with more categories; likelihood-ratio test *p*-value was > 0.05). Complete case analysis was used as fewer than 5% of observations had missing data [25] and multiple imputation for missing covariate data did not change overall findings. We obtained robust standard errors for estimating 95% confidence intervals (CIs) that account for clustering at the township level.

After storing RRR estimates for factors obtained from the observed data, the mlogit model was used to investigate how EBF prevalence would change in response to hypothetical changes in infants' hospital experiences. Specifically, we tested what would have happened to the distribution of current feeding groups under four simulated in-hospital scenarios informed by BFHI guidelines: 1) 75% of infants in the sample had received no formula in-hospital while the other 25% had received formula (water feeds unchanged); 2) 100% had received no formula (water feeds unchanged), 3) 100% had received no water (formula feeds unchanged), and 4) 75% had been exclusively breastfed (received no formula or water) and the other 25% had received formula but no water. This last scenario was chosen to represent criteria used for designating hospitals as “Baby-Friendly.” For each scenario, we first changed infants’ formula and/or water in-hospital feeding experience to match the hypothetical percentages for these practices (e.g., formula feeds were set to zero for all infants in the second scenario). All other factors, including feeding colostrum and early initiation of breastfeeding were left unchanged. Next, the stored RRR estimates from the mlogit model were used to calculate predicted probabilities of infants in each current feeding group under each of the hypothetical feeding scenarios, conditional on the remaining factors [26]. Standard errors for constructing 95% CIs on the predicted probabilities were calculated using the delta method [27]. The observed distribution of current feeding groups and the estimated distributions of current feeding groups in response to the four hypothetical scenarios were plotted as a series of five stacked bars. Each bar includes all children in the analytic sample and either their observed or hypothetical current feeding group membership (i.e., total equals 100%).

The 25% of infants who were to receive formula in hypothetical scenarios 1 and 4 were selected based on their predicted probability of actually having received formula in-hospital (see the Supplemental Materials for details). With this approach, infants with high probabilities of having received formula in the hospital in the observed data remain more likely to receive formula in the simulation compared to those with a low probability, thereby accounting for the likelihood of individual compliance with the hypothetical intervention.

All analyses were performed using Stata version 14.2 statistical software [24]. The code is reproduced in the Supplemental Materials.

## Results

### Sample characteristics

Of the 785 mother-infant dyads included in our analysis, mothers’ median age was 27 years and 16% had attained an educational level of tertiary or above (Table 1). About 78% of mothers had out-migrated previously. Infants were about three months old on average, 56.1% were born by C-section, and 63.5% were born at a county-level hospital. Nearly half (46.5%) of infants were fed breastmilk or colostrum as their first feed, although only 22.2% were breastfed within the first hour. Most infants (87.8%) were fed colostrum, 61.8% fed water or sugar water, and 77.6% fed formula at any time while in the hospital. Of those born by C-section, only 7.5% were EBF during their hospital stay (compared to 17.7% of those born by vaginal delivery), with 40.9% having received formula and 20.7% water as their first feed. Only five caregivers (0.64%) reported receiving an infant formula sample in a hospital or clinic. Formula feeding in-hospital was comparable among mothers who had and had not migrated for work previously (78.0% and 76.1%, respectively).

**Table 1:** Descriptive statistics for the full sample and by current feeding group membership

	Total	EBF	BF&L	MF	BF&S	NBF
	n (%)	n (%)	n (%)	n (%)	n (%)	n (%)
	785	299 (38.1)	151 (19.2)	192 (24.5)	59 (7.5)	84 (10.7)
**Infant characteristics**						
Infant age: 0 to < 2 m	293 (37.3)	127 (42.5)	49 (32.5)	92 (47.9)	8 (13.6)	17 (20.2)
Infant age: 2 to < 4 m	257 (32.7)	118 (39.5)	45 (29.8)	63 (32.8)	5 (8.5)	26 (31.0)
Infant age: 4 to < 6 m	235 (29.9)	54 (18.1)	57 (37.7)	37 (19.3)	46 (78.0)	41 (48.8)
Male	421 (53.6)	161 (53.8)	84 (55.6)	100 (52.1)	34 (57.6)	42 (50.0)
Vaginal birth	345 (43.9)	135 (45.2)	76 (50.3)	80 (41.7)	27 (45.8)	27 (31.2)
Born premature	30 (3.8)	9 (3.0)	3 (2.0)	10 (5.2)	3 (5.1)	5 (6.0)
Born low birth weight	29 (3.7)	6 (2.0)	7 (4.6)	11 (5.7)	2 (3.4)	3 (3.6)
**Maternal and family characteristics**						
Maternal age ≤ 27 y	399 (50.8)	159 (53.2)	85 (56.3)	88 (45.8)	31 (52.5)	36 (42.9)
Maternal age > 27 y	386 (49.2)	140 (46.8)	66 (43.7)	104 (54.2)	28 (47.5)	48 (57.1)
Maternal education						
Primary or less	102 (13.0)	33 (11.0)	24 (15.9)	24 (12.5)	7 (11.9)	14 (16.7)
Secondary	558 (71.1)	218 (72.9)	106 (70.2)	143 (74.5)	43 (72.9)	48 (57.1)
Tertiary or above	125 (15.9)	48 (16.1)	21 (13.9)	25 (13.0)	9 (15.3)	22 (26.2)
Mother married from outside village	363 (46.2)	142 (47.5)	75 (49.7)	87 (45.3)	27 (45.8)	32 (38.1)
Mother migrated previously for work	609 (77.6)	235 (78.6)	115 (76.2)	144 (75.0)	50 (84.7)	65 (77.4)
Household wealth						
1^st^ quintile	154 (19.6)	63 (21.1)	34 (22.5)	35 (18.2)	9 (15.3)	13 (15.5)
2^nd^ quintile	178 (22.7)	62 (20.7)	44 (29.1)	42 (21.9)	11 (18.6)	19 (22.6)
3^rd^ quintile	151 (19.2)	62 (20.7)	28 (18.5)	35 (18.2)	12 (20.3)	14 (16.7)
4^th^ quintile	185 (23.6)	75 (25.1)	27 (17.9)	42 (21.9)	16 (27.1)	25 (29.8)
5^th^ quintile	117 (14.9)	37 (12.4)	18 (11.9)	38 (19.8)	11 (18.6)	13 (15.5)
**Community-level and hospital characteristics**						
County 1	171 (21.8)	68 (22.7)	41 (27.2)	36 (18.8)	9 (15.3)	17 (20.2)
County 2	181 (23.1)	62 (20.7)	27 (17.9)	55 (28.6)	16 (27.1)	21 (25.0)
County 3	200 (25.5)	80 (26.8)	26 (17.2)	51 (26.6)	17 (28.8)	26 (31.0)
County 4	233 (29.7)	89 (29.8)	57 (37.7)	50 (26.0)	17 (28.8)	20 (23.8)
Residence in town	261 (33.2)	96 (32.1)	41 (27.2)	66 (34.4)	25 (42.4)	33 (39.3)
Birthing location						
Township health center	102 (13.0)	37 (12.4)	29 (19.2)	22 (11.5)	7 (11.9)	7 (8.3)
County MCH hospital	269 (34.3)	110 (36.8)	60 (39.7)	60 (31.3)	16 (27.1)	23 (27.4)
County hospital	229 (29.2)	86 (28.8)	33 (21.9)	60 (31.3)	25 (42.4)	25 (29.8)
City MCH hospital	43 (5.5)	16 (5.4)	3 (2.0)	11 (5.7)	4 (6.8)	9 (10.7)
City hospital	136 (17.3)	50 (16.7)	23 (15.2)	38 (19.8)	7 (11.9)	18 (21.4)
Other	6 (0.8)	0 (0.0)	3 (2.0)	1 (0.5)	0 (0.0)	2 (2.4)
**Feeding practices in hospital**						
Early breastfeeding initiation	174 (22.2)	69 (23.1)	42 (27.8)	35 (18.2)	16 (27.1)	12 (14.3)
First fed breastmilk/colostrum	365 (46.5)	162 (54.2)	78 (51.7)	69 (35.9)	25 (42.4)	31 (36.9)
First fed formula	265 (33.8)	89 (29.8)	46 (30.5)	76 (39.6)	18 (30.5)	36 (42.9)
First fed water, sugar water or other	155 (19.7)	48 (16.1)	27 (17.9)	47 (24.5)	16 (27.1)	17 (20.2)
Ever fed colostrum	689 (87.8)	274 (91.6)	134 (88.7)	168 (87.5)	55 (93.2)	58 (69.0)
Ever fed water	485 (61.8)	154 (51.5)	109 (72.2)	124 (64.6)	46 (78.0)	52 (61.9)
Ever fed formula	609 (77.6)	218 (72.9)	112 (74.2)	166 (86.5)	44 (74.6)	69 (82.1)

Categories of feeding in previous 24 h: *EBF* exclusively breastfed, *BF&L* fed breastmilk and non-milk liquids, *MF* mixed feeding of breastmilk and animal milk or formula, *BF&S* fed breastmilk and solid or semi-solid foods, *NBF* not breastfed, *MCH* Maternal and Child Health

Township health centers (THCs) typically do not have the authority to deliver babies, but some were likely former county-level hospitals that were re-categorized when townships were rezoned and were permitted to deliver babies for the convenience of nearby residents

Overall, city and county hospitals had better feeding practices; 23–27% of newborns experienced early breastfeeding initiation compared to only 7% in township-level hospitals (Table S5). However, formula feeds were common across all hospital types (~ 68–81% of newborns). Infants of the wealthiest families were more likely to have been born in city and county hospitals.

**Fig. 1:**
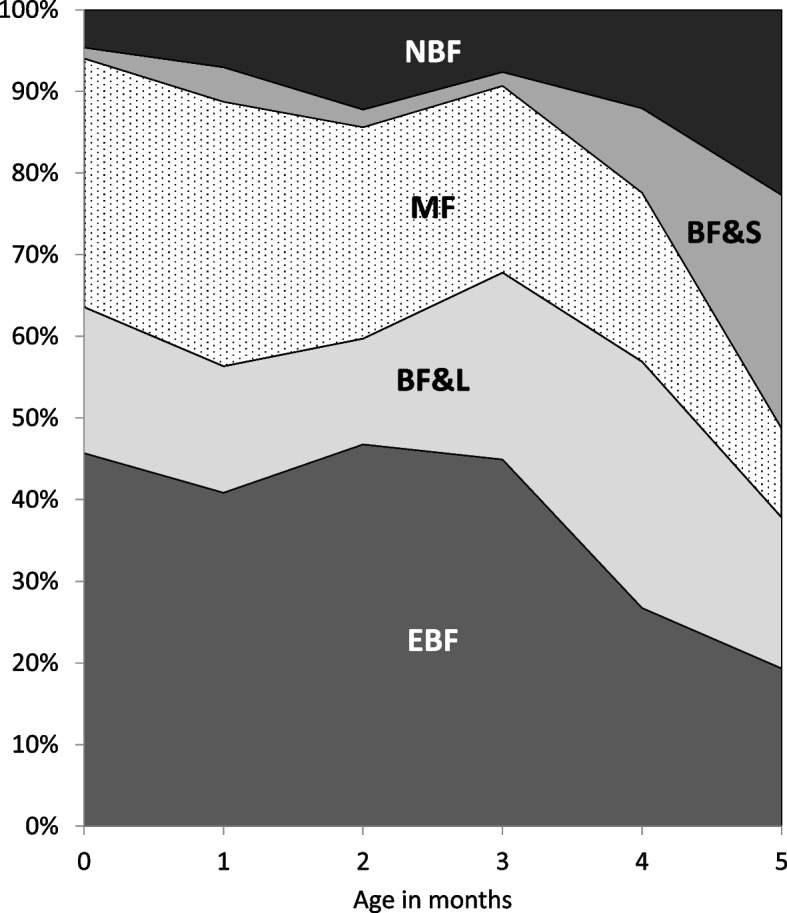
Distribution of prevalence of feeding practices as a function of infant age in months. Categories of feeding in previous 24 h: EBF, exclusively breastfed; BF&L, fed breastmilk and non-milk liquids; MF, mixed feeding of breastmilk and animal milk or formula; BF&S, fed breastmilk and solid or semi-solid foods; NBF, not breastfed. Note: Prevalence values are averages for age increments of 0 to < 1 m, 1 to < 2 m, 2 to < 3 m, 3 to < 4 m, 4 to < 5 m, 5 to < 6 m

Based on 24-h recall at the time of the survey, 38.1% of infants were exclusively breastfed, 19.2% were fed breastmilk with water or other non-milk liquid, 24.5% received mixed feeding, 7.5% were fed breastmilk with semi-solids or solids, and 10.7% were not breastfed. Figure 1 shows the distribution of current feeding group prevalence as a function of infant age in months, and indicates that there was a steep drop in EBF prevalence at 4–5 months of age.

### Associations between in-hospital postpartum and current feeding practices

Multinomial logistic regression results are presented in Table 2 for 761 mother-infant pairs. Twenty four pairs (~ 3% of sample) were excluded from the regression due to missing data for infant sex (*n* = 10), infant preterm status (*n* = 2), early breastfeeding initiation (*n* = 7), or because the infant was born at home or in an unknown setting (*n* = 5). Consistent with the pattern of decreasing EBF prevalence with age shown in Fig. 1, infants 4–5 months of age were more likely than 0–1-month-old infants to be fed breastmilk and non-milk liquids (RRR: 2.72, 95% CI 1.54, 4.79), breastmilk and animal milk or formula (RRR: 13.6, 95% CI 5.29, 35.0), or not breastfed (RRR: 6.30, 95% CI 3.08, 12.9) as opposed to EBF (Table 2).

**Table 2:** Adjusted multinomial logistic regression results for predictors of current feeding group membership

	BF&L RRR (95% CI)	MF RRR (95% CI)	BF&S RRR (95% CI)	NBF RRR (95% CI)
**Feeding practices in hospital**				
Early breastfeeding initiation	1.55 (0.96–2.51)	0.88 (0.53–1.47)	1.34 (0.69–2.57)	0.85 (0.38–1.91)
Ever fed colostrum	0.72 (0.37–1.39)	0.76 (0.41–1.43)	1.14 (0.35–3.72)	0.23*** (0.11–0.46)
Ever fed water	2.48*** (1.61–3.82)	1.59* (1.10–2.29)	3.33** (1.45–7.64)	1.36 (0.79–2.36)
Ever fed formula	1.03 (0.61–1.73)	2.52** (1.44–4.42)	1.04 (0.49–2.22)	1.76 (0.74–4.20)
**Infant characteristics**				
Infant age: 0 to < 2 m	1.00	1.00	1.00	1.00
Infant age: 2 to < 4 m	0.95 (0.58–1.56)	0.69 (0.45–1.07)	0.73 (0.25–2.12)	1.39 (0.72–2.70)
Infant age: 4 to < 6 m	2.72*** (1.54–4.79)	0.87 (0.50–1.54)	13.6*** (5.29–35.0)	6.30*** (3.08–12.9)
Male	1.24 (0.84–1.82)	0.93 (0.64–1.35)	1.37 (0.77–2.45)	0.83 (0.48–1.43)
Vaginal birth	1.25 (0.80–1.94)	1.28 (0.85–1.92)	1.39 (0.71–2.71)	0.94 (0.49–1.78)
Born premature or low birth weight	1.09 (0.41–2.89)	1.65 (0.75–3.61)	2.26 (0.70–7.25)	0.90 (0.25–3.22)
Maternal age^a^	1.00 (0.95–1.05)	1.06** (1.02–1.10)	1.00 (0.92–1.07)	1.07* (1.01–1.13)
Maternal education				
Primary or less	1.00	1.00	1.00	1.00
Secondary	0.80 (0.38–1.66)	0.96 (0.52–1.79)	0.79 (0.27–2.30)	0.61 (0.28–1.32)
Tertiary or above	0.81 (0.32–2.02)	0.70 (0.31–1.61)	0.86 (0.24–3.13)	1.28 (0.51–3.22)
Mother migrated previously for work	0.87 (0.52–1.45)	0.73 (0.45–1.20)	1.27 (0.58–2.80)	0.80 (0.42–1.54)
Household wealth				
1^st^ quintile	1.00	1.00	1.00	1.00
2^nd^ quintile	1.53 (0.80–2.93)	1.31 (0.74–2.29)	1.11 (0.44–2.82)	1.75 (0.65–4.71)
3^rd^ quintile	0.83 (0.41–1.68)	1.08 (0.59–1.95)	1.06 (0.40–2.76)	1.11 (0.41–3.03)
4^th^ quintile	0.70 (0.34–1.46)	0.98 (0.51–1.89)	0.78 (0.27–2.30)	1.08 (0.41–2.88)
5^th^ quintile	1.12 (0.54–2.36)	2.06* (1.15–3.70)	0.85 (0.24–3.04)	1.09 (0.36–3.29)
County 1	1.00	1.00	1.00	1.00
County 2	0.81 (0.44–1.50)	1.92* (1.05–3.50)	1.29 (0.42–4.03)	1.45 (0.67–3.09)
County 3	0.50* (0.26–0.99)	1.35 (0.74–2.48)	0.86 (0.26–2.82)	1.26 (0.53–3.01)
County 4	1.05 (0.57–1.94)	1.43 (0.79–2.58)	0.86 (0.26–2.82)	1.05 (0.44–2.51)
Residence in town	0.93 (0.59–1.47)	1.19 (0.76–1.85)	2.22* (1.14–4.34)	1.68 (0.94–3.00)
Birthing location				
Township health center	1.00	1.00	1.00	1.00
County hospital	0.90 (0.47–1.73)	1.26 (0.71–2.22)	1.71 (0.66–4.41)	1.64 (0.52–5.22)
City hospital	0.57 (0.31–1.47)	1.93* (1.01–3.07)	1.00 (0.35–2.83)	2.83 (0.96–8.29)

Categories of feeding in previous 24 h: *EBF* exclusively breastfed, *BF&L* fed breastmilk and non-milk liquids, *MF* mixed feeding of breastmilk and animal milk or formula, *BF&S* fed breastmilk and solid or semi-solid foods, *NBF* not breastfed

Adjusted multinomial logistic regression (mlogit) coefficients on predictor variables were estimated for each of five feeding groups relative to EBF, the reference group. Coefficients were exponentiated for ease of interpretation as relative risk ratios (RRR)

EBF was used as the reference outcome group

^*^ *p* < 0.05, ** *p* < 0.01, *** *p* < 0.001

^a^Maternal age was the only continuous variable in the regression (other variables were all categorical)

Compared to infants who were EBF, infants fed any water postpartum while in-hospital were 2.48 (95% CI 1.61, 3.82) times as likely as those who were not fed water to be fed breastmilk and non-milk liquids later, 1.59 (95% CI 1.10, 2.29) times as likely to receive mixed feeding, and 3.33 (95% CI 1.45, 7.64) times as likely to be fed breastmilk and solid or semi-solid foods. Infants fed any formula in-hospital were 2.52 (95% CI 1.44, 4.42) times as likely as those who were not to later receive mixed feeding compared to infants who were EBF. Associations with early breastfeeding initiation were not statistically significant for any non-EBF groups. Being fed colostrum was associated with a reduced RRR (0.23, 95% CI 0.11, 0.46) of not being breastfed compared to EBF.

### Hypothetical changes to current feeding group prevalence from changing in-hospital postpartum feeding practices

**Fig. 2:**
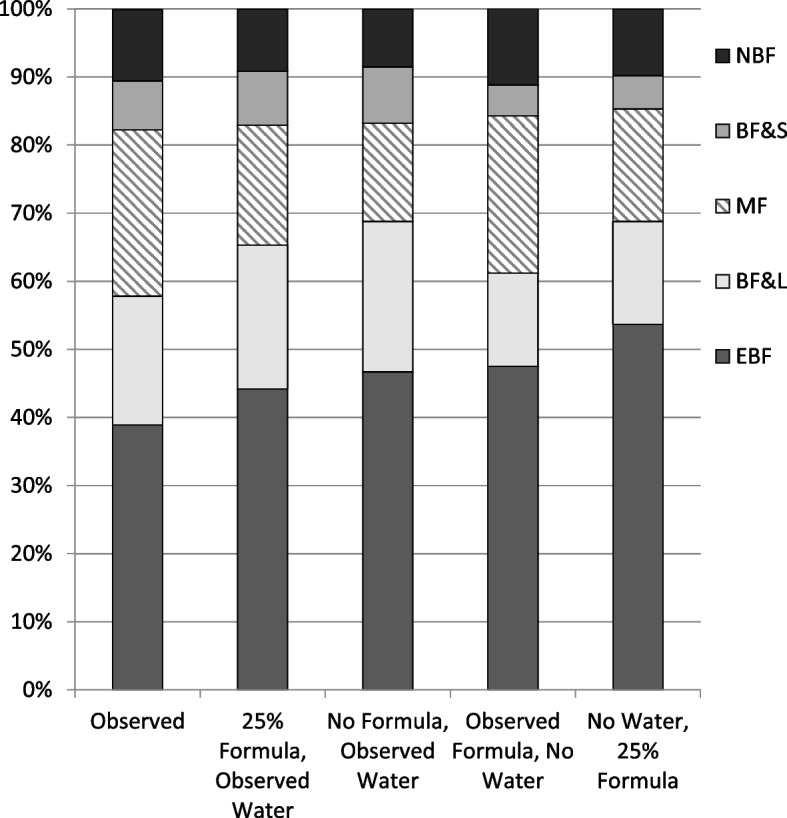
Distribution of infant feeding groups as observed and as estimated in response to hypothetical changes in hospital feeding practices. Categories of feeding in previous 24 h: EBF, exclusively breastfed; BF&L, fed breastmilk and non-milk liquids; MF, mixed feeding of breastmilk and animal milk or formula; BF&S, fed breastmilk and solid or semi-solid foods; NBF, not breastfed. Notes: The stacked bars represent the distribution of current feeding groups under five separate scenarios for hospital feeding practices: observed (first bar) and in response to one of the four simulated interventions. Each bar represents all children in the analytic sample and either their observed or hypothetical current feeding group membership (i.e., total equals 100%). To set 25% of the sample to receive formula, infants’ probability of receiving formula was predicted using logistic regression and the same factors used to explain current feeding practice. These probabilities were then scaled such that the overall average was 25%. Infants’ simulated formula feeding experience was drawn from a Bernoulli distribution with probability equal to their down-scaled probability

The percentages of infants by current feeding group are depicted in Fig. 2 for observed plus four simulated in-hospital feeding scenarios. By setting 75% of infants to not receive formula while in the hospital (25% formula), EBF membership increased from an estimated 38.9% (95% CI 35.7, 42.1) of infants to an estimated 44.2% (95% CI 38.2, 50.0), while mixed feeding membership dropped from an estimated 24.4% (95% CI 21.4, 27.4) to an estimated 17.6% (95% CI 13.5, 21.7). EBF membership increased to 46.7% (95% CI 38.7, 54.7) under the scenario of no formula feeds. In the absence of any water feeds (no water), EBF membership was estimated at 47.5% (95% CI 42.2, 52.9), while mixed feeding remained essentially unchanged. The combination of 75% exclusively breastfed newborns and no water feeds for any infants at discharge (no water, 25% formula) resulted in an estimated EBF membership of 53.7% (95% CI 46.1, 61.2).

## Discussion

In a sample of infants born in rural areas of Sichuan, China, about one-third (38%) of infants under six months of age had been exclusively breastfed at the time of data collection. Nearly 80% had been fed infant formula and nearly two-thirds (62%) were fed water or sugar water while still in the hospital postpartum. Both practices are well-known to be detrimental to later EBF [8, 10, 14] and our results support this evidence. However, empirical evidence is lacking for the impact that hospital compliance with newborn feeding guidelines could have on continued EBF in rural China. To address this gap, we first modeled the association of newborns’ in-hospital feeding experiences with current feeding practices, finding that infants who had been fed any water or formula while in-hospital were 2–3 times as likely to be non-EBF compared to EBF up to age six months. Using this model, we simulated alternate in-hospital feeding experiences and estimated the potential impact of changing in-hospital experiences at discharge on EBF prevalence during the first six months.

Importantly, our simulation results suggest that EBF prevalence could be improved meaningfully if in-hospital feeding practices were modified to reflect BFHI targets. In a hypothetical scenario specifying that 75% of infants were exclusively breastfed and no water-based feeds were given during the in-hospital postpartum period, the prevalence of infants exclusively breastfed to six months of age was estimated to increase from 38.9% (95% CI 35.7, 42.1) to 53.7% (95% CI 46.1, 61.2), a 14.8 absolute percentage point and a 38.0% relative improvement. The simulations also suggest that the elimination of in-hospital water-based feeds alone has the potential to increase later EBF prevalence by an estimated 8.6 absolute percentage points, principally by reducing the prevalence of infants later fed water and other non-milk liquids along with breastmilk. These findings are consistent with evidence [28], including from urban China [29], that infants whose feeding experiences align with BFHI guidelines are more likely to be EBF later. The results of our simulations extend this evidence to the context of rural Sichuan and suggest that promotion of BFHI guidelines may be an effective approach to increasing EBF rates, warranting consideration for policy implementation.

Fewer than half of mothers in the sample reported breastmilk or colostrum as their infant’s first feed with only 22.4% initiating breastfeeding within the first hour. Delays in breastfeeding initiation are likely attributable, in part, to the high percentage (56%) of births by C-section in our study and misinformation suggesting that women are unable to breastfeed immediately after the procedure [11, 30]. Given that only 7.5% of infants born by C-section were EBF while in the hospital, improving healthcare provider training to support early breastfeeding initiation, even after a C-section, and implementing policies to discourage non-medically indicated C-sections, could limit breastfeeding delays associated with the surgery. Further research is warranted to explore attitudes that may reflect a desire for more “medicalized” care among women undergoing non-medically indicated C-section.

The prevalence of postpartum formula use in our sample was high (≥ 68%) across all hospital types. Past migration history—our proxy for future migration and its potential influence on later infant feeding practices, for example, due to lack of designated times and places for breastfeeding in the workplace —was not associated with infants’ in-hospital feeding experiences; the percent of infants who were fed formula postpartum was similar irrespective of mother’s history of migration. Formula marketing in hospitals in China has been described in previous research [1, 31]; however, only a few women in our study reported being offered free formula samples in the hospital after childbirth. This self-reported result may have been influenced by social desirability bias, but also does not preclude formula marketing via other channels from being a possible influencing factor on their infant feeding practices. While our study did not explicitly examine the reasons for using infant formula in the hospital, the universally high rates regardless of demographic and hospital characteristics suggest that perceptions and practices around formula use may be normative. Similarly, reasons for the high prevalence of feeding newborns water or sugar water (~ 62%) are unknown in our study. As there are no medically indicated reasons for water-based feeds, these practices may also be driven by cultural beliefs and social norms, as well as by family-based misinformation, as reported elsewhere [16, 32]. While research has shown that social norms are often difficult to change, interventions explicitly targeting norms, and that engage multiple stakeholders through multiple mechanisms, can successfully transform harmful normative practices [33, 34]. Thus, alongside policies promoting BHFI feeding practices, it may be necessary to introduce social norms interventions, such as strengthening sanctions against marketing of infant formula or educating families that the benefits of EBF assume elimination of water-based feeds.

The major limitation of our study was that the data were cross-sectional, having been collected for another purpose. Temporality concerns associated with cross-sectional data were minimized by focusing on factors that occurred prior to the survey (e.g., during the in-hospital postpartum period) or were non-time varying (e.g., maternal education). However, unmeasured confounding from factors that were not assessed prior to the child’s birth, such as feeding preferences and perceived familial social support for breastfeeding, may have biased our results and limit our ability to make causal claims. This study also had possible recall bias of hospital experiences, and possible misclassification of feeding group membership, which was based on feeding during the 24 h prior to the survey rather than routinely. The use of 24-h recall to assess infant feeding is widely accepted for surveys, including UNICEF’s Multiple Indicator Cluster Surveys [22]. However, a longer recall period (e.g., 3–7 days) may be more accurate and warranted in future research. We did not collect information on the length of mothers’ hospital stay, which could also bias our findings if the length of stay was associated with hospital feeding experiences and systematically different by current feeding group. We aimed to minimize this source of bias by excluding infants who were not breastfed due to medical reasons. Differences between excluded households in child age, sex, and household wealth may be a source of selection bias. Importantly, we cannot generalize our findings to the experience of all infants in rural China, although our results are consistent with research in different regions of China including urban settings [11, 14, 15].

## Conclusions

Given the importance of an infant’s first feeding experiences in the establishment and continuation of EBF, it is imperative that rural Chinese hospitals actively seek to reduce infant formula feeds to medically indicated situations and eliminate water-based feeds. Our results suggest that infant feeding outcomes would improve substantially if rural hospitals met BFHI guidelines for EBF from birth to discharge. The significance of infant first feed in the hospital has been underappreciated, understudied, and overlooked as an important target of intervention to improve EBF in China. Transforming infants’ first feeding experiences in rural China will likely require normative change, driven by national and local public health leadership, to address misinformation around best practices. Additional research is needed to improve our understanding of the role of families and social norms in influencing feeding practices during the postpartum period and how hospital policy or practices might be harnessed to address harmful normative practices. Our results also suggest the need for further examination of the role of C-section and length of hospital stay on hospital feeding experiences, as well as additional qualitative research to understand choices in infant feeding practices in rural China.

## Supplementary Information

**Additional file 1.**

## Data Availability

Data are available upon reasonable request.

## Abbreviations

BFHI: Baby-Friendly Hospital Initiative
CI: Confidence interval
C-section: Cesarean section
EBF: Exclusive breastfeeding
LMIC: Low- and middle-income countries
MCH: Maternal and Child Health
THC: Township health center
mlogit: Multinomial logistic
RRR: Relative risk ratio
WHO: World Health Organization
