# Supplementary material for: Associations of in-hospital postpartum feeding experiences with exclusive breastfeeding practices among infants in rural Sichuan, China
Source: Int Breastfeed J. 2023 Jul 13;18:34. doi: 10.1186/s13006-023-00567-z (PMC10347832; doi:10.1186/s13006-023-00567-z)
Supplement: Supplementary file 1 — Additional file 1. [file 13006_2023_567_MOESM1_ESM.docx]

**SUPPLEMENTAL MATERIALS**

**Multinomial logistic (mlogit) regression**

Mlogit is an extension of logistic regression that is used when the outcome has more than two unordered categories, such as our six current feeding groups. The exclusive breastfeeding (EBF) group was selected as the reference category for purposes of identifiability. In logistic regression, the estimated coefficients in the model have a natural interpretation as log odds ratios. They are often exponentiated for ease of interpretation as odds ratios. However, in mlogit models, a different set of coefficients is estimated for each group relative to the reference group, which, when exponentiated, have a natural interpretation as relative risk ratios (RRR).

To understand the use of these terms,^[[1]](#footnote-1)^ consider that if we had only one binary predictor variable (e.g., male/female), the mlogit model would estimate eight coefficients, the intercept and the coefficient on the predictor for each of the four non-EBF categories. For example, the coefficients on sex would be:

exp(b1)=[P(BF&L)/P(EBF) | male] / [P(BF&L)/P(EBF) | female] (1)

exp(b2)=[P(MF)/P(EBF) | male] / [P(MF)/P(Y=EBF) | female] (2)

for BF&L (1) and MF (2), and so on for the other three feeding categories. Each equation can be re-written to see that they represent a ratio of two relative risks (or RRR), for example:

exp(b2)=[P(MF |male)/ P(MF|female)] / [P(EBF|male)/ P(EBF|female)] (3)

**Identification of 25% of infants intervened on to receive formula**

To identify which 25% of infants were to receive formula in the simulation scenarios 1 and 4, we first predicted infants’ probability of having received formula in-hospital with logistic regression conditional on the same predictor variables used to explain their current feeding practice. Next, probabilities of formula feeds were scaled down such that the overall average for the sample was 25%. Infants’ simulated formula feeding experience (received or not) was then drawn from a Bernoulli distribution with probability equal to their down-scaled probability.

**Stata Code**

*============================================================*

*

* In-hospital postpartum feeding experiences and EBF in rural China

* Code for mlogit regression and marginal means under hypothetical scenarios

* Author: Ann Weber

* Date: March 4, 2022

*

*============================================================*

* The following code is for data from a cross-sectional survey of moms of infants 0-6 months of age

* The outcome variables is a 5-level current feeding practices variables, cfp_n5, with EBF as the reference category

* Global sets of covariates used in mlogit and logit commands

global fpract2 "i.bf_earlyinit i.fed_colostrum i.hspfed_anywater i.hspfed_formula"

global demog2 "i.age_cat2 child_sex m_age i.m_edu2 i.mom_mig_befor i.hh_qtile i.vil_town i.county"

global deliv "i.deliv_location2 i.deliv_method i.lbw_prem"

* Series of indicator variables for multi-category variables for SE calculations were

set more off

foreach var of varlist deliv_location2 age_cat2 m_edu2 hh_qtile county {

tab `var', gen(d`var')

}

global myvars "ddeliv_location22 ddeliv_location23 deliv_method lbw_prem bf_earlyinit fed_colostrum hspfed_anywater hspfed_formula dage_cat22 dage_cat23 child_sex m_age dm_edu22 dm_edu23 mom_mig_befor dhh_qtile2 dhh_qtile3 dhh_qtile4 dhh_qtile5 vil_town dcounty2 dcounty3 dcounty4"

************

* Author written programs for calculating predicted probabilities and SEs when setting 25% of infants to receive formula (with and without water).

* Adapted from UCLA: Statistical Consulting Group webpage: <https://stats.oarc.ucla.edu/stata/code/manually-generate-predicted-probabilities-from-a-multinomial-logistic-regression-in-stata/>

* And from the Stata FAQs: <https://www.stata.com/support/faqs/statistics/standard-error-predicted-probability/> and <https://www.stata.com/support/faqs/statistics/delta-method/>

* Obtain SE for category 1 - EBF

gen one = 1

cap program drop cat1

program define cat1, rclass

* Gradient for category 1 (1 row, columms)

foreach n of numlist 2/5 {

local i=0

gen p1p`n' = p1*p`n'

foreach var of global myvars {

local i = `i' + 1

gen p1p`n'x`i' = p1p`n'*`var'

}

}

sum p1p*

mkmat one, matrix(U)

mkmat p1p2 - p1p5x23, matrix(P1)

mat S1 = U'*P1

mat grad = -S1/rowsof(P1)

mat grad1 = grad[1,2..24],grad[1,1],grad[1,26..48],grad[1,25],grad[1,50..72],grad[1,49],grad[1,74..96],grad[1,73]

* Get SE

matrix Vx1 = grad1*Zz*grad1'

mean p1 p2 p3 p4 p5

disp sqrt(Vx1[1,1])

return local se1 = sqrt(Vx1[1,1])

end

* Obtain SE for category 3 - MF

cap program drop cat3

program define cat3, rclass

* Gradient for category 3 (1 row, columms)

foreach n of numlist 2 4/5 {

gen p3p`n' = p3*p`n'

local j=0

foreach var of global myvars {

local j = `j' + 1

gen p3p`n'x`j' = p3p`n'*`var'

}

}

gen p3po3 = p3*(1-p3)

local i=0

foreach var of global myvars {

local i = `i' + 1

gen p3po3x`i' = p3po3*`var'

}

mkmat p3p2 - p3p2x23, matrix(P3a)

mkmat p3po3 - p3po3x23, matrix(P3b)

mkmat p3p4 - p3p5x23, matrix(P3c)

mat S3a = U'*P3a

mat S3b = U'*P3b

mat S3c = U'*P3c

mat grad3a = -S3a/rowsof(P3a)

mat grad3b = S3b/rowsof(P3b)

mat grad3c = -S3c/rowsof(P3c)

mat grad = grad3a,grad3b,grad3c

mat grad3 = grad[1,2..24],grad[1,1],grad[1,26..48],grad[1,25],grad[1,50..72],grad[1,49],grad[1,74..96],grad[1,73]

* Get SE

matrix Vx3 = grad3*Zz*grad3'

mean p1 p2 p3 p4 p5

display sqrt(Vx3[1,1])

return local se1 = sqrt(Vx3[1,1])

end

***************

* Run mlogit regression (adjusted for clustering at township level) and obtain RRR for Table 2

set more off

qui: mlog cfp_n5 $deliv $fpract2 $demog2, vce(cluster township)

mlogit, rrr

estimates store m1

* Obtain marginal means under different hypothetical scenarios and SEs from Stata margins command for no water or no formula

* Observed

margins, post

estimates restore m1

* Set formula to 0%

margins, at(hspfed_formula=0) post

estimates restore m1

* Set water to 0%

margins, at(hspfed_anywater=0) post

* Set formula to 25%

* Obtain marginal means and SEs from author-written code for setting 25% of infants to formula and 75% no formula (with and without water)

preserve

set more off

* predict formula feeding

qui: logit hspfed_form $demog2 $deliv i.bf_earlyinit i.fed_colostrum i.hspfed_anywater, vce(cluster township)

predict pf

sum pf /* .7757732 */

disp 0.25/r(mean)

* Multiply their pform by 0.32 (0.25/0.77) to get a target marginal probability of formula use of 25% - pf_target

gen pf_target = pf*(0.25/r(mean))

sum pf_target

* Run mlogit

estimates restore m1

*qui: mlog cfp_n5 $deliv $fpract2 $demog2, vce(cluster township)

qui: estat vce

matrix Z = r(V)

matrix Zz = Z[25..120,25..120]

* Intervene to set formula to 25%

set seed 834649587

drop hspfed_formula

gen hspfed_formula = runiform() < pf_target

tab hspfed_formula

predict p1 p2 p3 p4 p5 if e(sample)

cat1

* Calculate and return std errors that will be used to calculate CI's

return list

cat3

return list

restore

* Set formula to 25% & water to 0

preserve

set more off

* predict formula feeding

qui: logit hspfed_form $demog2 $deliv i.bf_earlyinit i.fed_colostrum i.hspfed_anywater, vce(cluster township)

predict pf

sum pf /* .7757732 */

disp 0.25/r(mean)

* Multiply their pform by 0.32 (0.25/0.77) to get a target marginal probability of formula use of 25% - pf_target

gen pf_target = pf*(0.25/r(mean))

sum pf_target

* Run mlogit

estimates restore m1

* qui: mlog cfp_n5 $deliv $fpract2 $demog2, vce(cluster township)

qui: estat vce

matrix Z = r(V)

matrix Zz = Z[25..120,25..120]

* Intervene to set formula to 25% (75% no formula) and water to 0%

recode hspfed_anywater 1=0

set seed 834649587

drop hspfed_formula

gen hspfed_formula = runiform() < pf_target

tab hspfed_formula

predict p1 p2 p3 p4 p5 if e(sample)

* Calculate and return std errors that will be used to calculate CI's

cat1

return list

cat3

return list

restore

**Tables**

**Table S1.** Food Types used to Generate Current Feeding Practice Categories

| **Food Type** | **Definition** | **Survey Questions^[[2]](#footnote-2)^** |
| --- | --- | --- |
| 1. Breastmilk | Breastmilk produced by mothers. | Was the child breastfed yesterday during the day or at night? |
| 2. Breastmilk substitutes (BMS) | Formula, animal milks such as cow's milk, or other types of milk used to mimic or replace breastmilk. | Did the child have any infant formula yesterday? |
|  |  | Did the child have any milk such as tinned, powdered, or fresh animal milk yesterday? |
| 3. Thin liquids | Thin liquids used mainly for additional hydration rather than a main source of energy. | Did the child have any plain water yesterday? |
|  |  | Did the child have any juice or juice drink yesterday? |
|  |  | Did the child have any clear broth yesterday? |
| 4. Semi-solid and solid foods | Calorie dense foods that provide energy to the infant in addition to milk. | Did the child have any yogurt yesterday? |
|  |  | Did the child have any thin porridge yesterday? |
|  |  | Did the child have any other liquid yesterday (e.g., sugar water, soda, tea, soymilk)? |
|  |  | At what age did the baby start to have complementary foods (liquid, semisolid, or solid foods other than breastmilk or formula that provide nutrients)? * |
|  |  | Did the baby eat any staple food yesterday, such as rice porridge, flour porridge, steamed bun or rice |
|  |  | Did the baby eat any yellow or orange food yesterday, such as pumpkin, carrot or red sweet potato |
|  |  | Did the baby eat any root and stem vegetables yesterday, such as potato, yam, radish, white sweet potato |
|  |  | Did the baby eat any leafy dark green vegetables yesterday? |
|  |  | Did the baby eat any red or yellow fruits such as persimmon, apricot, watermelon, cantaloupe or tomato yesterday? |
|  |  | Did the baby eat any organ meats such as animal liver, kidney or heart yesterday? |
|  |  | Did the baby eat any other meat or meat products (e.g., chicken, duck, pork, beef, lamb, etc.) yesterday? |
|  |  | Did the baby eat any eggs yesterday? |
|  |  | Did the baby eat any fresh or dried fish, shellfish or seafood yesterday? |
|  |  | Did the baby eat any beans, peas, lentils, nuts or seeds yesterday? |
|  |  | Please note: Drinking soymilk counts |
|  |  | Did the baby eat any dairy products such as cheese and yoghurt yesterday? |

- Answer to each of the questions was coded into 0 (no), or 1 (yes)
- *If respondent answered, "The baby has not started complementary foods yet", they received a score of zero and did not answer the subsequent questions

**Table S2.** Current Feeding Practice Categories

| **Categories (5)** | **Breastmilk** | **Water or other non-milk liquids** | **Breastmilk substitute** | **Semi-solid or solid foods** |
| --- | --- | --- | --- | --- |
| Exclusively breastfed (EBF) | yes | no | no | no |
| Breastfed and non-milk liquids (BF&L) | yes | yes | no | no |
| Breastfed and formula or animal milk (mixed feeding or MF) | yes | maybe | yes | no |
| Breastfed and solid foods (BF&S) | yes | maybe | maybe | yes |
| Not breastfed (NBF) | no | maybe | maybe | maybe |

- Breastmilk substitutes include formula, animal milks such as cow's milk, or other types of milk used to mimic or replace breastmilk
- Non-milk liquids include liquids used mainly for additional hydration rather than a main source of energy, including water, sugar water, juice and broth.
- Semi-solid and solid foods are complementary foods that provide energy to the infant in addition to milk, for example porridge or rice.

**Table S3. Survey questions^[[3]](#footnote-3)^ for in-hospital feeding practices after birth**

| **Survey Questions** | **Answer choices** |
| --- | --- |
| Has the child ever been breastfed? | 1=yes  2=no |
| How soon after birth did the baby suckle at the breast for the first time?  *[If respondent reports she put the infant to the breast in less than 1 hour, circle ‘1’ for hours AND RECORD ‘00’ hours. If less than 24 hours, circle ‘1’ and record number of completed hours, from 01 to 23. Otherwise, circle ‘2’ and record number of completed days.]* | 1 = hours \|__\|__\|  2 =days  \|__\|__\|  3 = never  999 = don’t know |
| What was the child fed first after birth? | 1 = breastmilk/colostrum  2 = formula  3 = water  4 = other (specify)______  999 = don’t know |
| Was the child fed colostrum? | 1 = yes  2 = no  999 = don’t know |
| Was the baby fed water at any time in the hospital? | 1 = yes  2 = no  999 = don’t know |
| Was the baby fed sugared water at any time in the hospital? | 1 = yes  2 = no  999 = don’t know |
| Was the baby fed formula at any time in the hospital? | 1 = yes  2 = no  999 = don’t know |
| From where have you received infant formula samples? | 1 = hospital/clinic  2 = baby store  3 = supermarket  4 = gift from friends/neighbors/family  5 = other, specify_____  6 = have never received any infant formula sample |

**Table S4. Descriptive characteristics and hospital feeding practices by inclusion status**

|  | Included | Excluded | p-value |
| --- | --- | --- | --- |
|  | (n=785) | (n=45) |  |
| **Infant characteristics** |  |  |  |
| Infant age: 0 to <2 m | 293 (37.3%) | 9 (20.0%) | 0.004 |
| Infant age: 2 to <4 m | 257 (32.7%) | 11 (24.4%) |  |
| Infant age: 4 to <6 m | 235 (29.9%) | 23 (51.1%) |  |
| Male | 421 (53.6%) | 15 (33.3%) | 0.013 |
| Vaginal birth | 345 (43.9%) | 19 (42.2%) | 0.92 |
| Born premature | 30 (3.8%) | 5 (11.1%) | 0.013 |
| Born low birth weight | 29 (3.7%) | 3 (6.7%) | 0.30 |
| **Maternal and family characteristics** |  |  |  |
| Caregiver age > median (27 y) | 386 (49.2%) | 29 (64.4%) | 0.046 |
| Caregiver education |  |  |  |
| Primary or less | 102 (13.0%) | 6 (13.3%) | 0.77 |
| Secondary | 558 (71.1%) | 24 (53.3%) |  |
| Tertiary | 125 (15.9%) | 5 (11.1%) |  |
| Household wealth |  |  |  |
| 1st quintile | 154 (19.6%) | 22 (48.9%) | <0.001 |
| 2^nd^ quintile | 178 (22.7%) | 9 (20.0%) |  |
| 3^rd^ quintile | 151 (19.2%) | 8 (17.8%) |  |
| 4^th^ quintile | 185 (23.6%) | 4 (8.9%) |  |
| 5^th^ quintile | 117 (14.9%) | 2 (4.4%) |  |
| Primary caregiver is biological mother | 785 (100.0%) | 9 (20.0%) | <0.001 |
| Mother married from outside village | 422 (53.8%) | 4 (8.9%) | 0.39 |
| Mother migrated previously for work | 609 (77.6%) | 8 (17.8%) | 0.86 |
| **Community-level and hospital characteristics** | | | |
| County |  |  |  |
| County 1 | 171 (21.8%) | 12 (26.7%) | 0.67 |
| County 2 | 181 (23.1%) | 11 (24.4%) |  |
| County 3 | 200 (25.5%) | 8 (17.8%) |  |
| County 4 | 233 (29.7%) | 14 (31.1%) |  |
| Town residence | 261 (33.2%) | 13 (28.9%) | 0.55 |
| Birthing location |  |  | 0.16 |
| Township health center | 102 (13.0%) | 5 (11.1%) |  |
| County MCH hospital | 269 (34.3%) | 15 (33.3%) |  |
| County hospital | 229 (29.2%) | 7 (15.6%) |  |
| City MCH hospital | 43 (5.5%) | 3 (6.7%) |  |
| City hospital | 136 (17.3%) | 14 (31.1%) |  |
| Other | 6 (0.8%) | 0 (0.0%) |  |
| **Feeding practices in hospital** |  |  |  |
| Early breastfeeding initiation | 174 (22.2%) | 4 (8.9%) | 0.065 |
| Breastmilk/colostrum | 365 (46.5%) | 10 (22.2%) | 0.008 |
| Formula | 265 (33.8%) | 22 (48.9%) |  |
| Water/sugar water/other | 155 (19.7%) | 12 (26.7%) |  |
| Ever fed colostrum | 689 (87.8%) | 26 (57.8%) | <0.001 |
| Ever fed water | 485 (61.8%) | 28 (62.2%) | 0.81 |
| Ever fed formula | 609 (77.6%) | 37 (82.2%) | 0.31 |

- MCH = Maternal and Child Health
- Data are presented as n (%)

**Table S5.** Descriptive characteristics and feeding practices in the hospital by birthing location

|  | Township Health Center | County MCH Hospital | County General Hospital | City MCH Hospital | City General Hospital | Other | p-value |
| --- | --- | --- | --- | --- | --- | --- | --- |
|  | (n=102) | (n=269) | (n=229) | (n=43) | (n=136) | (n=6) |  |
| **Infant characteristics** | | | | | | | |
| Infant age | | | | | | | |
| 0 to <2 m | 35 (34.3) | 107 (39.8) | 79 (34.5) | 21 (48.8) | 50 (36.8) | 1 (16.7) | 0.77 |
| 2 to <4 m | 33 (32.4) | 86 (32.0) | 81 (35.4) | 12 (27.9) | 43 (31.6) | 2 (33.3) |  |
| 4 to <6 m | 34 (33.3) | 76 (28.3) | 69 (30.1) | 10 (23.3) | 43 (31.6) | 3 (50.0) |  |
| Male | 48 (47.1) | 141 (52.4) | 135 (59.0) | 27 (62.8) | 67 (49.3) | 3 (50.0) | 0.21 |
| Vaginal birth | 52 (51.0) | 126 (46.8) | 89 (38.9) | 15 (34.9) | 60 (44.1) | 3 (50.0) | 0.23 |
| Born premature | 3 (2.9) | 9 (3.3) | 6 (2.6) | 2 (4.7) | 9 (6.6) | 1 (16.7) | 0.23 |
| Born low birth weight | 5 (4.9) | 7 (2.6) | 6 (2.6) | 3 (7.0) | 7 (5.1) | 1 (16.7) | 0.22 |
| **Maternal and family characteristics** | | | | | | | |
| Maternal age | | | | | | | |
| ≤ median (27 y) | 50 (49.0) | 144 (53.5) | 109 (47.6) | 23 (53.5) | 68 (50.0) | 5 (83.3) | 0.47 |
| > median (27 y) | 52 (51.0) | 125 (46.5) | 120 (52.4) | 20 (46.5) | 68 (50.0) | 1 (16.7) |  |
| Maternal education | | | | | | | |
| Primary or less | 16 (15.7) | 42 (15.6) | 25 (10.9) | 8 (18.6) | 10 (7.4) | 1 (16.7) | 0.16 |
| Secondary | 78 (76.5) | 183 (68.0) | 166 (72.5) | 29 (67.4) | 98 (72.1) | 4 (66.7) |  |
| Tertiary | 8 (7.8) | 44 (16.4) | 38 (16.6) | 6 (14.0) | 28 (20.6) | 1 (16.7) |  |
| Household wealth | | | | | | | |
| 1st quintile | 27 (26.5) | 62 (23.0) | 32 (14.0) | 11 (25.6) | 22 (16.2) | 0 (0.0) | 0.022 |
| 2^nd^ quintile | 27 (26.5) | 58 (21.6) | 47 (20.5) | 10 (23.3) | 34 (25.0) | 2 (33.3) |  |
| 3^rd^ quintile | 24 (23.5) | 49 (18.2) | 42 (18.3) | 12 (27.9) | 23 (16.9) | 1 (16.7) |  |
| 4^th^ quintile | 15 (14.7) | 61 (22.7) | 64 (27.9) | 9 (20.9) | 33 (24.3) | 3 (50.0) |  |
| 5^th^ quintile | 9 (8.8) | 39 (14.5) | 44 (19.2) | 1 (2.3) | 24 (17.6) | 0 (0.0) |  |
| Mother married from outside village | 51 (50.0) | 121 (45.0) | 98 (42.8) | 19 (44.2) | 70 (51.5) | 4 (66.7) | 0.49 |
| Mother migrated previously for work | 77 (75.5) | 207 (77.0) | 174 (76.0) | 37 (86.0) | 108 (79.4) | 6 (100.0) | 0.49 |
| **Community-level characteristics** | | | | | | | |
| County | | | | | | | |
| County 1 | 38 (37.3) | 29 (10.8) | 8 (3.5) | 24 (55.8) | 69 (50.7) | 3 (50.0) | <0.001 |
| County 2 | 24 (23.5) | 26 (9.7) | 65 (28.4) | 15 (34.9) | 51 (37.5) | 0 (0.0) |  |
| County 3 | 14 (13.7) | 72 (26.8) | 100 (43.7) | 3 (7.0) | 9 (6.6) | 2 (33.3) |  |
| County 4 | 26 (25.5) | 142 (52.8) | 56 (24.5) | 1 (2.3) | 7 (5.1) | 1 (16.7) |  |
| Town residence | 41 (40.2) | 77 (28.6) | 78 (34.1) | 15 (34.9) | 49 (36.0) | 1 (16.7) | 0.29 |
| **Feeding practices in hospital** | | | | | | | |
| Early breastfeeding initiation | 7 (6.9) | 68 (25.3) | 53 (23.1) | 10 (23.3) | 36 (26.5) | 0 (0.0) | 0.002 |
| First fed after birth | | | | | | | |
| Breastmilk/colostrum | 20 (19.6) | 143 (53.2) | 92 (40.2) | 26 (60.5) | 84 (61.8) | 0 (0.0) | <0.001 |
| Formula | 37 (36.3) | 103 (38.3) | 79 (34.5) | 12 (27.9) | 31 (22.8) | 3 (50.0) |  |
| Water/sugar water/other | 45 (44.1) | 23 (8.6) | 58 (25.3) | 5 (11.6) | 21 (15.4) | 3 (50.0) |  |
| Ever fed colostrum | 89 (87.3) | 239 (88.8) | 206 (90.0) | 35 (81.4) | 116 (85.3) | 4 (66.7) | 0.28 |
| Ever fed water | 87 (85.3) | 129 (48.0) | 159 (69.4) | 24 (55.8) | 83 (61.0) | 3 (50.0) | <0.001 |
| Ever fed formula | 80 (78.4) | 218 (81.0) | 182 (79.5) | 32 (74.4) | 93 (68.4) | 4 (66.7) | 0.086 |
| - MCH = Maternal and Child Health - Data are presented as n (%) | | | | | | | |

1. Adapted from Stata blog explanation at <https://www.stata.com/statalist/archive/2005-06/msg00069.html> [↑](#footnote-ref-1)
2. Survey questions are adapted from WHO, UNICEF. *Indicators for Assessing Infant and Young Child Feeding Practices: Definitions and Measurement Methods*.; 2021. [https://www.who.int/publications-detail-redirect/9789240018389](https://nam04.safelinks.protection.outlook.com/?url=https%3A%2F%2Fwww.who.int%2Fpublications-detail-redirect%2F9789240018389&data=04%7C01%7Cannweber%40unr.edu%7C48fc0fbcdd7f4ed5a30008d9fb162b6b%7C523b4bfc0ebd4c03b2b96f6a17fd31d8%7C1%7C0%7C637816894924631118%7CUnknown%7CTWFpbGZsb3d8eyJWIjoiMC4wLjAwMDAiLCJQIjoiV2luMzIiLCJBTiI6Ik1haWwiLCJXVCI6Mn0%3D%7C3000&sdata=i2ewaLdTEUH7rUZBwktWXaYoxmxlDN9puKF03XpSHZY%3D&reserved=0) [↑](#footnote-ref-2)
3. First two questions are adapted from WHO, UNICEF. *Indicators for Assessing Infant and Young Child Feeding Practices: Definitions and Measurement Methods*; 2021. [https://www.who.int/publications-detail-redirect/9789240018389](https://nam04.safelinks.protection.outlook.com/?url=https%3A%2F%2Fwww.who.int%2Fpublications-detail-redirect%2F9789240018389&data=04%7C01%7Cannweber%40unr.edu%7C48fc0fbcdd7f4ed5a30008d9fb162b6b%7C523b4bfc0ebd4c03b2b96f6a17fd31d8%7C1%7C0%7C637816894924631118%7CUnknown%7CTWFpbGZsb3d8eyJWIjoiMC4wLjAwMDAiLCJQIjoiV2luMzIiLCJBTiI6Ik1haWwiLCJXVCI6Mn0%3D%7C3000&sdata=i2ewaLdTEUH7rUZBwktWXaYoxmxlDN9puKF03XpSHZY%3D&reserved=0). The remaining questions are study authors’ questions based on their previous research. [↑](#footnote-ref-3)
